# Computational identification of putative mRNA-miRNA signatures in chemoresistant and metastatic triple-negative breast cancer

**DOI:** 10.1016/j.bbrep.2026.102656

**Published:** 2026-06-04

**Authors:** Mahvash Farajzadeh-Dehkordi, Elham Rezvani Boroujeni, Sina Darzi, Manijeh Jalilvand, Babak Rahmani

**Affiliations:** a Department of Molecular Medicine, Qazvin University of Medical Sciences, Qazvin, Iran; b Cellular and Molecular Research Center, Institute for Prevention of Non-Communicable Diseases, Qazvin University of Medical Sciences, Qazvin, Iran; c Department of Microbiology and Microbial Biotechnology, Faculty of Life Sciences and Biotechnology, Shahid Beheshti University, Tehran, Iran; d Health Products Safety Research Center, Qazvin University of Medical Sciences, Qazvin, Iran; e Department of Biochemistry and Genetics, Cellular and Molecular Research Center, Qazvin University of Medical Sciences, Iran

## Abstract

Triple-negative breast cancer (TNBC) is a heterogeneous malignancy that is recognized as having a high rate of metastasis and limited treatment options. Identifying new molecular signatures for TNBC remains urgent and challenging. In this study, we aimed to identify novel mRNA and miRNA expression profiles associated with invasive and drug-resistant TNBC using bioinformatics methods. We analyzed four datasets (GSE43502, GSE88847, GSE61723, and GSE154255) obtained from the Gene Expression Omnibus (GEO) repository. Significantly differentially expressed genes (DEGs) and miRNAs (DEMs), along with mRNA-miRNA interaction networks, were identified, visualized, and validated using *R* packages, enrichment analysis, protein-protein interaction (PPI) networks, and in silico validation through the UALCAN database. A total of 3242 DEGs and 95 DEMs were identified. Among these DEMs, 18 (13 downregulated and 5 upregulated) were selected as novel candidates. Target genes related to the candidate miRNAs were predicted and overlapped with DEGs. We identified 111 commonly upregulated and 129 commonly downregulated genes. Gene set enrichment analysis revealed that upregulated genes were involved in immune-related pathways, whereas downregulated genes were enriched in oncogenic signaling pathways, including MAPK and BRAF/RAF1 fusions. The mRNA-miRNA network highlighted miR-4690-5p and miR-338-3p as key regulators. *In silico* validation results confirmed that the BRD4 and LYN genes, as well as miR-16-2-3p, were significantly overexpressed, while the XBP1 gene and miR-5684 were markedly downregulated in TNBC samples. Overall, this study reveals computationally novel mRNA and miRNA expression profiles associated with metastatic and chemoresistant TNBC samples. These findings provide preliminary molecular insights may serve as tissue-specific and context-dependent biomarkers for future experimental investigations.

## Highlights

• Novel mRNA-miRNA signatures in aggressive TNBC identified.
• 18 novel miRNA candidates proposed from multi-dataset analysis.
• Immune pathway activation and MAPK fusions found in TNBC.
• MiR-4690-5p and miR-338-3p emerge as key regulatory hubs.
• Validation confirms *BRD4*, *LYN*, and miR-16-2-3p overexpression.

## Graphical abstract

**Figure:**
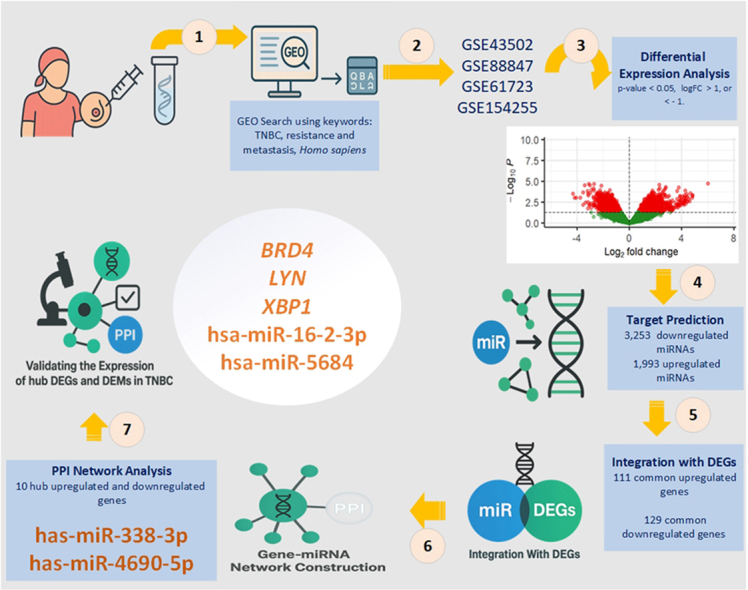

## 1 Introduction

Worldwide, breast cancer (BC) is one of the most prevalent solid tumors in women and represents the second leading cause of cancer-related deaths among females each year [1]. Triple-negative breast cancer (TNBC), a major subtype of BC, accounts for 15-25% of all invasive cases and is clinically defined by the lack of amplification of the human epidermal growth factor receptor 2 (HER2), progesterone receptor (PR), and estrogen receptor (ER).TNBC is a heterogeneous malignancy at the clinical, phenotypic, histological, and molecular levels [2,3].

It is well known that TNBC cells exhibit distinct biological characteristics, including a high degree of histological malignancy, large size, rapid proliferation, and aggressive metastatic tumors, which are associated with the poorest prognosis and lowest survival rates compared to other BC subtypes [4]. Evidence indicates that TNBC is mainly detected in females at a relatively young age, and most patients experience a high recurrence rate [5]. Despite advances in therapeutic targets and biomarker discovery for BC patients in recent decades, TNBC lacks specific receptor markers in clinical practice, rendering patients ineligible for molecular-targeted therapies and endocrine treatments [6]. As a result, non-specific chemotherapy remains the only systematic treatment option for non-surgical TNBC cases in both early and metastatic stages. Although TNBC patients initially respond favorably to conventional chemotherapy, resistance often develops rapidly. There is an urgent need for novel therapeutic biomarkers to improve prognosis, treatment strategies, and enhance the survival of TNBC cases [7,8].

In recent years, genomic technologies such as high-throughput sequencing, transcriptomics, and microarray approaches have become increasingly valuable in clinical research [[9], [10], [11], [12]]. These methods provide robust strategies for identifying key genes involved in the initiation and progression of carcinogenesis, while also providing valuable opportunities to discover novel targets [13,14]. However, the predictive power of single-gene biomarkers often varies across studies due to limited sample sizes and the heterogeneous nature of carcinogens. Moreover, such biomarkers may fail to accurately reflect the complex gene network underlying cancer development [15].

Therefore, a comprehensive analysis of large-scale genomic data is essential for obtaining reliable and reproducible results. At present, numerous studies on TNBC drug resistance have concentrated on identifying genes, mRNA, miRNAs, and their interaction using bioinformatics approaches [16,17]. However, a complete understanding of the underlying molecular mechanisms remains elusive. The aim of this study was to identify putative mRNA-miRNA regulatory signatures associated with metastatic and chemoresistant TNBC through integrative bioinformatics analysis of publicly available transcriptomic datasets. We anticipated that our findings would provide a solid scientific basis for recognizing potential targets to enhance the diagnosis, treatment, and prognosis of TNBC patients.

## 2 Materials and methods

### 2.1 Extraction of data from the GEO database

To collect suitable TNBC microarray datasets that included both gene and miRNA profiles, we retrieved data from the Gene Expression Omnibus (GEO, https://www.ncbi.nlm.nih.gov/geo/). The inclusion criteria comprised the terms "TNBC", "resistance/recurrence/relapse/metastasis", and "adjacent normal tissues", with the organism restricted to *Homo sapiens*, study type defined as " expression profiling by array", and a minimum sample size of more than 20. Based on these criteria, three gene expression datasets (GSE43502, GSE88847, and GSE61723) and one miRNA dataset (GSE154255) were selected. In GSE43502 (25 samples), 16 TNBC patients with tumor recurrence after neoadjuvant chemotherapy were compared with nine patients without recurrence following a similar regimen. In GSE88847 (37 samples), six TNBC patients with recurrence were compared to 31 patients without recurrence. In GSE61723 (65 samples), 48 TNBC patients with metastasis were compared with 17 patients without metastasis. In another clinical analysis, GSE 154255 (20 samples),10 BC patients were compared with 10 normal breast tissue samples.

### 2.2 Identification of DEGs and DEMs

Differentially expressed genes (DEGs) and differentially expressed microRNAs (DEMs) in TNBC were identified using R software (version 4.4.1) together with several packages, such as limma (3.48.3), data.table (1.14.2), plyr (1.8.6), BiocGenerics (0.40.0), BioBase (2.54), and ggplot2 (3.3.5). The EnhancedVolcano package was additionally applied to generate volcano plots. Screening criteria were set to adjusted p-value (FDR) < 0.05, and absolute log2 fold change (|log_2_FC|) ≥ 1. P-values were adjusted for multiple testing using the default Benjamini -Hochberg false discovery rate method. To further enhance the robustness of the finding, only overlapping DEGs consistently identified across the four independent datasets were retained for subsequent analyses.

### 2.3 Prediction of target genes for DEMs

Following the identification of novel DEMs, this list was further refined to include only candidates with no previous reported association with TNBC, based on publicly available literature resources. This curated set was then used for target prediction analysis. To predict the targets for candidate DEMs, the MultiMiR R package (version 1.14.0) was employed. Subsequently, data obtained from multiMiR and DEGs identified from three datasets (GSE43502, GSE88847, and GSE61723) were entered into the Venn diagram web tool (https://bioinformatics.psb.ugent.be/webtools/Venn) to visualize the interaction between DEM-predicted target genes and DEGs.

### 2.4 Gene set enrichment analysis

To evaluate the biological relevance of overlapping DEGs, gene set enrichment analysis was conducted through the Enrichr web server (http://amp.pharm.mssm.edu/Enrichr) [18].

### 2.5 Protein-Protein Interaction (PPI) analysis, gene -miRNA network construction

The STRING database (http://string.embl.de/) [19], together with Cytoscape software (version 3.7.2) [20] were applied to build and visualize PPI networks among shared DEGs. The CytoHubba plugin (version 0.1) in Cytoscape was used to identify central node genes, which were also considered top hub genes for further analysis. The mRNA-miRNA interaction network was also illustrated using Cytoscape.

### 2.6 In silico validation of hub DEGs and DEMs in TNBC

Expression profiles of candidate DEMs and hub genes in TNBC clinical samples were examined using the in silico UALCAN platform (https://ualcan.path.uab.edu/index.html) [21], applying a significance threshold of p-value <0.05.

## 3 Results

### 3.1 Identification of DEGs and DEMs

Differential expression analysis was performed on three datasets (GSE43502, GSE61723, and GSE88847). Specifically, 3125 DEGs were identified in GSE43502, including 1536 upregulated and 1589 downregulated genes. In GSE88847, 39 DEGs were detected, comprising 35 upregulated and 4 downregulated genes. In GSE61723, 78 DEGs were found, with 34 upregulated and 44 downregulated genes (Supplementary File 1). Additionally, differential expression analysis of miRNAs in GSE154255 revealed a total of 95 DEMs, including 43 upregulated and 52 downregulated miRNAs (Supplementary File 1).

### 3.2 Prediction of target genes for DEMs

From the 95 DEMs identified, 18 DEMs (13 downregulated and 5 upregulated miRNAs) showed no previously reported association with TNBC, based on a literature search of publicly available sources. Therefore, we selected these miRNAs as novel candidate DEMs for target gene prediction (Table 1).

**Table 1:** List of 18 novel candidate DEMs with no previously reported association with TNBC. DEMs = Differentially expressed miRNAs.

miRNA - ID	logFC	P-value	adj. P. Val	Down/Up Regulation
hsa-miR-3137	−2.857977906	0.001848106	0.0138818	Down
hsa-miR-3125	−2.835892425	0.001726816	0.0138818	Down
hsa-miR-4481	−2.815848927	0.000207262	0.012992761	Down
hsa-miR-6512-5p	−2.719125142	0.002034345	0.0138818	Down
hsa-miR-125b-2-3p	−2.539480743	0.004495298	0.0138818	Down
hsa-miR-1288	−2.526337549	0.00506775	0.0138818	Down
hsa-miR-4707-5p	−2.44819259	0.006635197	0.0138818	Down
hsa-miR-4758-5p	−2.257225311	0.011185141	0.01727282	Down
hsa-miR-4716-3p	−2.09170436	0.017196763	0.026173526	Down
hsa-miR-1273e	−1.953879817	0.020401718	0.030817655	Down
hsa-miR-5684	−1.912630486	0.027066782	0.040549638	Down
hsa-miR-4690-5p	−1.674987891	0.03647085	0.053993008	Down
hsa-miR-5581-5p	−1.629121456	0.042539864	0.062562293	Down
hsa-miR-16-2-3p	1.074332827	0.034785897	0.051574656	UP
hsa-miR-338-3p	1.732774102	0.015146577	0.023140925	UP
hsa-let-7i-3p	1.915355115	0.031630418	0.047070192	UP
hsa-miR-4477a	1.977390744	0.024550279	0.036917437	UP
hsa-miR-491-3p	3.208209332	0.001673667	0.0138818	Up

To predict the target genes of novel candidate DEMs (13 downregulated and 5 upregulated miRNAs), the MultiMiR tool was used. This tool predicted 3253 target genes for downregulated miRNAs and 1993 for upregulated miRNAs (Supplementary File 2).

**Fig. 1:**
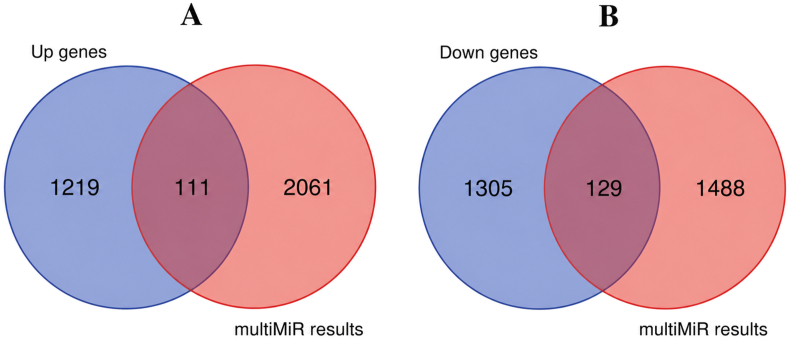
Enhancing biological relevance through integration of predicted miRNA targets with DEGs using volcano plots. **(A)** The volcano plot identified 111 common upregulated DEGs shared between upregulated DEGs and predicted target genes of corresponding DEMs. **(B)** The volcano diagram represented 129 common downregulated DEGs overlapping between the downregulated DEGs and the predicted target genes of corresponding DEMs. DEGs = differentially expressed genes; DEMs = differentially expressed miRNAs.

To enhance the biological relevance of the targets identified by MultiMiR, the predicted target genes of DEMs were overlapped with the DEGs list (up- and downregulated genes) from three datasets (GSE43502, GSE88847, and GSE61723) using the Volcano diagram marker. As illustrated in Fig. 1A, the overlap between upregulated DEGs and the predicted target genes of related DEMs resulted in 111 common upregulated genes (Supplementary File 2). Similarly, the Venn diagram in Fig. 1B represented the intersection of downregulated DEGs with predicted DEMs targets, resulting in 129 common downregulated genes (Supplementary File 2).

### 3.3 Gene set enrichment analysis

**Fig. 2:**
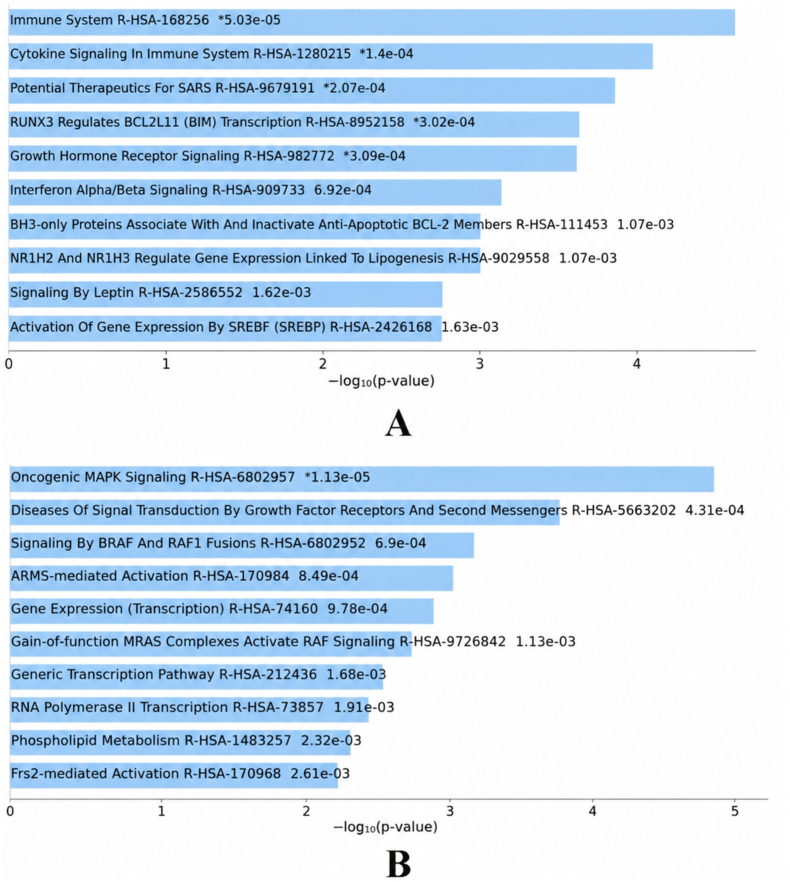
Pathway enrichment analysis performed using the online Enrich server. **(A)** Upregulated DEGs in TNBC were enriched in immune-related pathways, including “Immune system”, “Cytokine Signaling”, and “Potential Therapeutic for SARS”. **(B)** Downregulated DEGs were primarily associated with cancer-related signaling pathways, such as “MAPK signaling”, “Signal Transduction Disorders”, and “BRAF/RAF1 fusions”. DEGs = differentially expressed genes.

To explore the biological pathways associated with common GEGs in TNBC, enrichment analysis was carried out using the Enrich platform. The study indicated that 111 common upregulated DEGs were significantly enriched in pathways such as “Immune System” (p. value: 5.03E-05; Gene count: 25), “Cytokine Signaling in Immune System” (p. value: 1.40E-04; Gene count: 13), and “Potential Therapeutics For SARS” (p. value: 2.07E-04; Gene count: 5) (Fig. 2A). In contrast, 129 common downregulated DEGs were mainly related to “Oncogenic MAPK Signaling R-HSA” (p. value: 1.13E-05; Gene count: 6), “Diseases of Signal Transduction by Growth Factor Receptors and Second Messengers R-HSA” (p. value: 4.31E-04; Gene count: 10), and “Signaling by BRAF And RAF1 Fusions R-HSA” (p. value: 6.90 E−04; Gene count: 4) (Fig. 2B).

### 3.4 PPI network analysis, and Gene-miRNA network construction

**Fig. 3:**
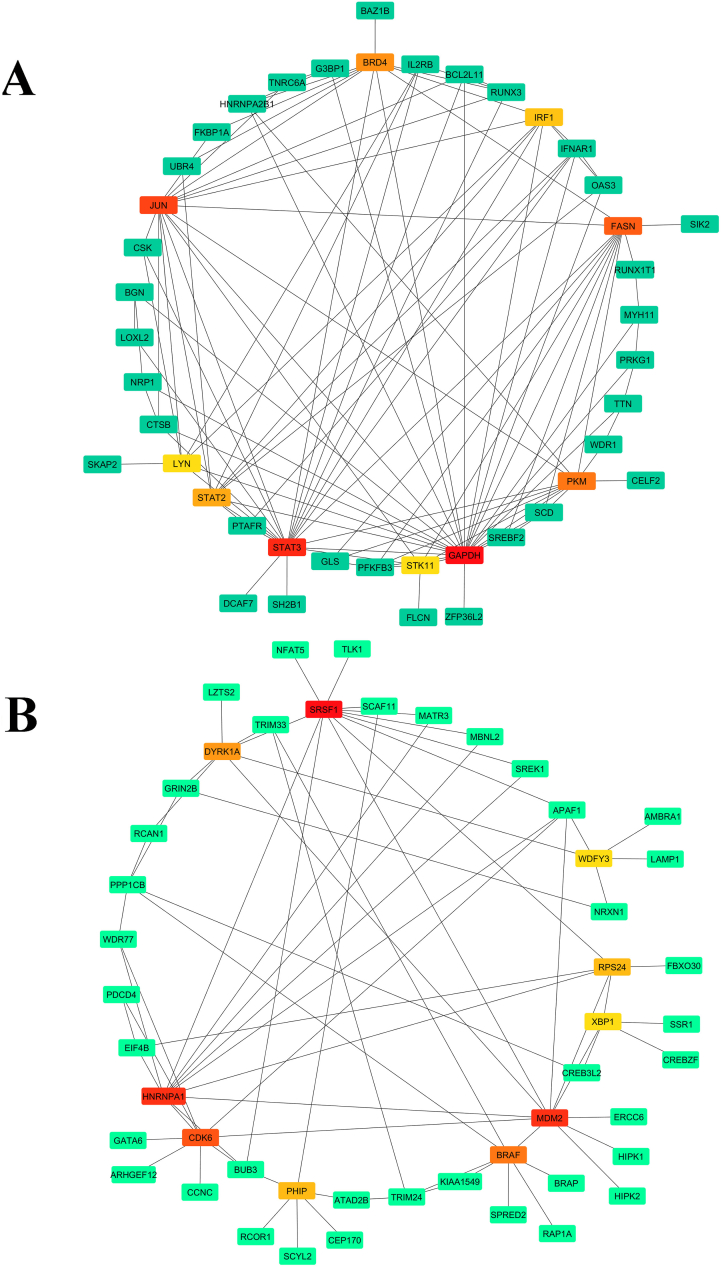
Construction and visualization of protein-protein interaction (PPI) networks using the STRING database and Cytoscape software. **(A)** PPI network of upregulated DEGs in TNBC showing 43 nodes and 106 edges. **(B)** PPI network of downregulated DEGs in TNBC, comprising 48 nodes and 5 edges. DEGs = differentially expressed genes.

**Fig. 4:**
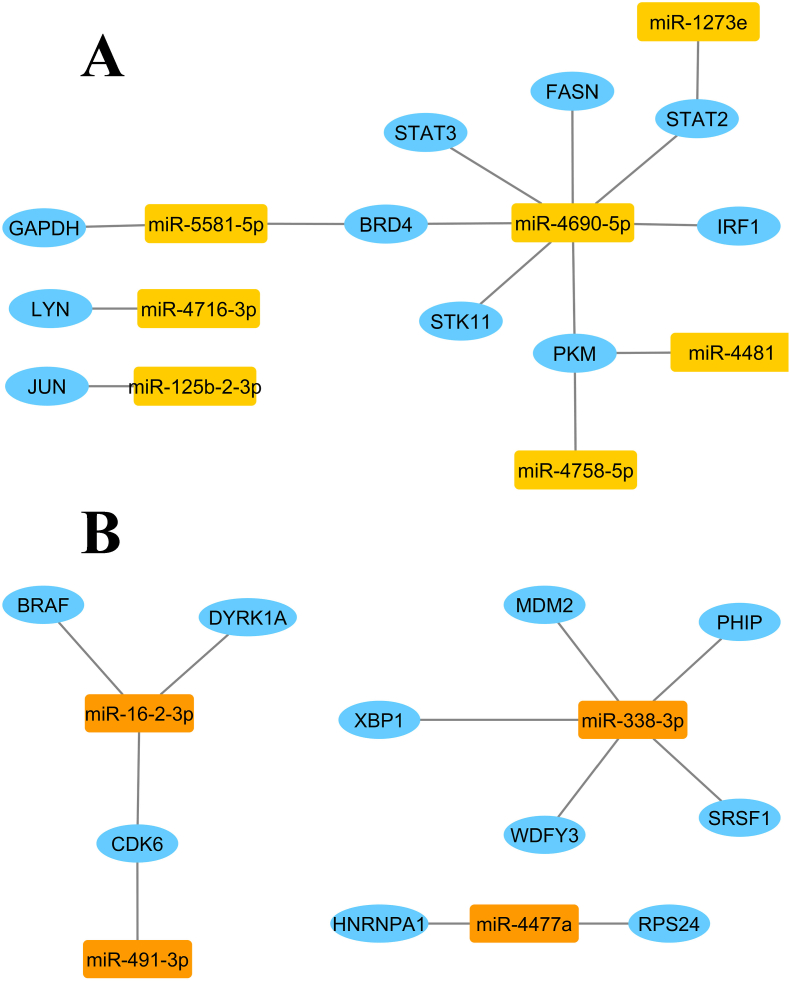
**(A)** Interaction network of upregulated hub genes and their predicted regulatory DEMs. Notably, miR-4690-5p was predicted to target seven hub genes, indicating a potential role in TNBC aggressiveness and chemoresistance. **(B)** Interaction network of downregulated hub genes and their predicted regulatory DEMs. Among these, has-miR-338-3p was predicted to target five hub genes, suggesting a possible role in TNBC chemoresistance and aggressiveness. DEMs = differentially expressed miRNAs.

A functional insight network of common DEGs involved in TNBC was constructed using the STRING database and visualized through Cytoscape. The PPI network derived from the upregulated DEGs consisted of 43 nodes and 106 edges (Fig. 3A). Subsequently, application of the CytoHubba plugin facilitated the identification of the top 10 upregulated hub genes: GAPDH, STAT3, JUN, FASN, PKM, BRD4, STAT2, IRF1, STK11, LYN, and BGN (Table 2). The network of these upregulated hub genes and related DEMs is illustrated in Fig. 4. These upregulated hub genes are predicted to be regulated by hsa -miR-5581-5p, hsa -miR-4690-5p, hsa -miR-125b-2-3p, hsa -miR-4758-5p, hsa -miR-4481, hsa -miR-1273e, and hsa -miR-4716-3p. Notably, hsa -miR-4690-5p was computationally predicted to target the most hub genes (n = 7), suggesting its potential involvement in TNBC aggressiveness and resistance to chemotherapeutic agents (Fig. 4A).

**Table 2:** Top 10 upregulated hub genes identified using the Degree method of the CytoHubba plugin.

Rank	Name	Ensemble	Score
1	GAPDH	ENSG00000108821	24
2	STAT3	ENSG00000164692	19
3	JUN	ENSG00000102265	15
4	FASN	ENSG00000118785	12
5	PKM	ENSG00000204262	11
6	BRD4	ENSG00000186340	10
7	STAT2	ENSG00000187498	9
8	IRF1	ENSG00000184956	8
9	STK11	ENSG00000169429	7
9	LYN	ENSG00000182492	7

In the context of downregulated DEGs, the PPI network comprised 48 nodes and 75 edges (Fig. 3B). Through application of the CytoHubba plugin, the top 10 hub downregulated genes, including SRSF1, MDM2, HNRNPA1, CDK6, BRAF, DYRK1A, RPS24, PHIP, XBP1, and WDFY3, were identified (Table 3). The integrative network of these hub genes and their related DEMs is depicted in Fig. 4B and Table 4. Bioinformatic predictions suggest that downregulated hub genes are potentially regulated by hsa-miR-338-3p, hsa -miR-4477a, hsa -miR-491-3p, and hsa -miR-16-2-3p. Among these candidates, hsa -miR-338-3p exhibited the highest number of hub genes (n = 5), implying a pivotal regulatory role in mediating TNBC aggressiveness and resistance in TNBC (Fig. 4B).

**Table 3:** Top 10 downregulated hub genes identified using the Degree method of the CytoHubba plugin.

Rank	Name	Ensemble	Score
1	SRSF1	ENSG00000136450	12
2	MDM2	ENSG00000135679	11
2	HNRNPA1	ENSG00000135486	11
4	CDK6	ENSG00000105810	9
5	BRAF	ENSG00000157764	8
6	DYRK1A	ENSG00000157540	7
7	RPS24	ENSG00000138326	6
7	PHIP	ENSG00000146247	6
9	XBP1	ENSG00000100219	5
9	WDFY3	ENSG00000163625	5

**Table 4:** Top Gene–miRNA interactions.

Gene	microRNA
GAPDH, BRD4	hsa-miR-5581-5p
STAT3, FASN, PKM, BRD4, STAT2, IRF1, STK11	hsa-miR-4690-5p
JUN	hsa-miR-125b-2-3p
STAT2	hsa-miR-1273e
PKM	hsa-miR-4481
LYN	hsa-miR-4716-3p
PKM	hsa-miR-4758-5p
CDK6, BRAF, DYRK1A	hsa-miR-16-2-3p
SRSF1, MDM2, PHIP, XBP1, WDFY3	hsa-miR-338-3p
HNRNPA1, RPS24	hsa-miR-4477a
CDK6	hsa-miR-491-3p

### 3.5 Validating the expression of hub DEGs and DEMs in TNBC

**Fig. 5:**
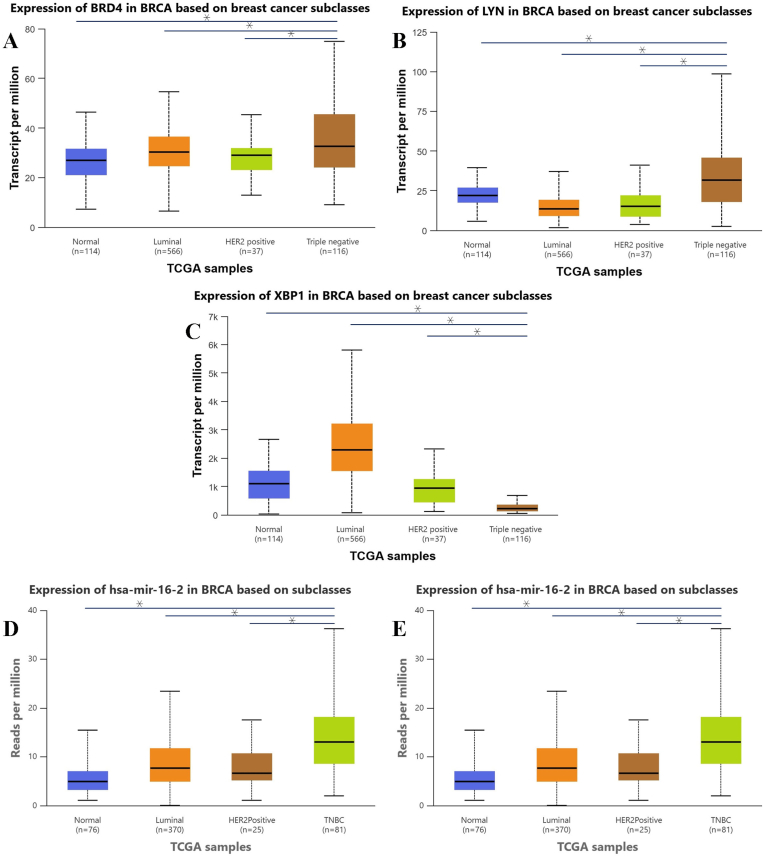
In silico validation of hub gene and candidate DEM expression in TNBC samples using the UALCAN database. **(A)** the upregulated hub gene BRD4 showed significantly higher mRNA expression in TNBC samples compared to non-TNBC subtypes and normal tissues. **(B)** the upregulated hub gene LYN exhibited significantly elevated mRNA expression in TNBC samples. **(C)** the downregulated hub gene XBP1 showed significantly reduced mRNA expression in TNBC samples relative to non-TNBC subtypes and normal tissues. **(D)** the upregulated candidate DEM hsa-miR-16-2-3p displayed significantly higher expression in TNBC samples. **(E)** the downregulated candidate DEM hsa-miR-5684 showed significantly lower expression in TNBC samples compared to non-TNBC subtypes and normal tissues. DEGs = differentially expressed genes, DEMs = differentially expressed miRNAs, TNBC = triple-negative breast cancer. ∗*P value* < 0.05.

To assess the transcriptional profile of the identified hub genes and candidate DEMs in clinical specimens from TNBC patients, the UALCAN online database (TCGA-derived expression derived) was used. This computational validation does not represent experimental conformation. Among the upregulated hub genes, BRD4 and LYN exhibited significantly elevated mRNA expression levels in TNBC tissues relative to non-TNBC subtypes and adjacent normal controls. (Fig. 5A and B). Conversely, within the downregulated hub genes set, XBP1 demonstrated significantly reduced mRNA expression in TNBC samples compared to both non-TNBC subtypes and normal breast tissues (Fig. 5C). Regarding upregulated candidate DEMs, hsa-miR-16-2-3p showed a significant increase in expression in TNBC samples, relative to non-TNBC and normal tissues (Fig. 5D). Among the downregulated candidate DEMs, hsa-miR-5684 displayed a pronounced decrease in expression in TNBC samples, when compared to non-TNBC subtypes and normal controls (Fig. 5E).

## 4 Discussion

TNBC, the most malignant subtype of BC, is recognized as having rapid metastasis, high invasiveness, poor differentiation, lack of specific therapeutic targets, and resistance to chemotherapy. In the present study, we conducted differential expression analysis to delineate hub genes and miRNAs potentially implicated in TNBC progression and acquired chemoresistance. Based on invasive and chemoresistant TNBC samples from the GEO database, we analyzed three mRNA datasets (GSE43502, GSE88847, and GSE61723) and one miRNA dataset (GSE154255). Our analysis revealed 1605 upregulated and 1637 downregulated genes. In addition, differential miRNA expression analysis identified 95 DEMs (43 up- and 52 downregulated miRNAs). Among 95 identified DEMs, 18 DEMs (13 downregulated and 5 upregulated miRNAs) showed no previously reported association with TNBC based on a literature review of publicly available sources. Thus, we selected them as novel candidate DEMs for target gene prediction. Using the MultiMiR tool, we identified 3253 target genes for downregulated candidate miRNAs and 1993 for upregulated candidate miRNAs. Overlapping DEGs between the predicted target genes of DEMs and those identified across the three datasets (GSE43502, GSE61723, and GSE88847), were then determined, resulting in 111 common upregulated and 129 common downregulated genes. Functional enrichment analysis demonstrated that upregulated DEGs were predominantly involved in the immune system, cytokine signaling, and anti-response signaling networks. In contrast, the downregulated DEGs were significantly enriched in oncogenic MAPK signaling, signal transduction disorders, and growth factor receptor pathways. To further investigate the target genes, we performed PPI network analysis and identified the top 10 upregulated hub genes, including GAPDH, STAT3, JUN, FASN, PKM, BRD4, STAT2, IRF1, STK11, LYN, and BGN. Similarly, the top 10 downregulated hub genes were SRSF1, MDM2, HNRNPA1, CDK6, BRAF, DYRK1A, RPS24, PHIP, XBP1, and WDFY3. Network analysis also highlighted hsa-miR-4690-5p and hsa-miR-338-3p as the most significant miRNAs in regulating upregulated and downregulated genes in TNBC, respectively.

Hsa-miR-4690-5p, a significantly downregulated miRNA, exhibited the highest number of upregulated target genes. The biological relevance of miR-4690 has been previously reported in various pathological contexts. Tűzesi Á et al. (2017) determined that hsa-miR-4690-5p was one of the top 10 downregulated miRNAs in the pediatric glioma stem cells compared to the normal neural stem cells [22]. Araki Y et al. (2023) identified hsa-miR-4690 as one of the miRNAs present in small extracellular vesicles released by high-grade metastatic osteosarcoma cells [23]. Akula SM et al. (2022) reported that the circulating level of hsa-miR-4690 was significantly altered in all individuals with COVID-19 [24]. Furthermore, Wang C et al. (2024) identified has-miR-4690 as one of the top 10 hub miRNAs potentially involved in fertilization processes [25]. Our findings indicate that downregulated miR-4690 is predicted to target multiple critical genes in oncogenic processes, immune regulation, and metabolic pathways. These observations suggest that miR-4690 may play a contributory role in promoting metastasis and drug resistance in TNBC; However, its precise functional involvement requires further experimental investigation. Based on the miRNA-mRNA interaction network analysis, hsa-miR-338-3p was identified as a significantly upregulated miRNA, exhibiting the highest number of predicted interactions with downregulated target genes. Previous studies have predominantly characterized miR-338 as a tumor suppressor in various malignancies by targeting genes and signaling pathways involved in tumor progression [[26], [27], [28]]. In BC tissue and cell lines, miR-338-3p has been reported to be downregulated and suppress tumor cell proliferation and increase apoptosis through ZEB2 and MORC4 genes targeting [29,30]. However, in the present study hsa-mir-338-3p was found to be upregulated in invasive and drug-resistant TNBC samples. In silico validation results from UALCAN further confirmed the elevated expression of miR-338-3p in TNBC compared to HER2-positive samples. Consistent with our findings, a number of studies have also reported hsa-miR-338 to be upregulated in various pathological conditions. Long J et al. (2018) reported that upregulation of miR-338-5p induced the proliferation and metastasis of melanoma cells via CD82 targeting and *p*-AKT upregulation [31]. Chu CA et al. (2019) reported that upregulation of miR-338-5p promoted colorectal cancer invasion by targeting PIK3C3, and elevated miR-338-5p expression was directly associated with advanced tumor stages and poor survival in colorectal cancer patients [32]. Lin L et al. (2024) demonstrated that upregulation of miR-338-3p attenuated the expression of apoptotic-related proteins and pro-inflammatory mediators induced by hypoxia/reoxygenation in myocardial infarction cell models [33]. Our results suggest that upregulated hsa-miR-338-3p may targets several key genes involved in tumor progression, cell cycle regulation, apoptosis, and stress response pathways. This pattern indicates that miR-338-3p may function as a context-dependent regulator in invasive and drug-resistant TNBC. Using the UALCAN tool, we assessed the expression level of the top 10 hub genes in TNBC samples. Among the upregulated genes, BRD4 and LYN showed markedly higher expression in TNBC compared to non-TNBC subtypes and normal tissue. Conversely, among downregulated hub genes, XBP1exhibited significantly lower expression in TNBC samples relative to non-TNBC subtypes and normal tissue.

Bromodomain-containing protein 4 (BRD4) plays a key role in gene transcription regulation, cell cycle progression, and the expression of key oncogenic driver genes [34,35]. Multiple studies have highlighted its involvement in immune cell infiltration, metastasis, and tumor progression in various tumors, including BC cells [[36], [37], [38], [39]]. Andrieu G et al. (2016) showed that BRD4 enhances the migration and invasion of TNBC cells by regulating Jagged1 expression [40]. Zhou JX et al. (2022) revealed that BRD4 promotes tumorigenesis in TNBC by modulating the transcription of mutant p53 [41]. In recent years, several therapeutic approaches targeting BRD4 in TNBC have emerged. Ali A et al. (2021) demonstrated that combined inhibition of BRD4-RAC1 signaling suppresses tumor growth in drug-resistant TNBC [42]. Similarly, Chi S et al. (2025) found that combining CDK4/6 and BRD4 inhibitors synergistically inhibits the growth of TNBC and ER-positive cells by inducing apoptosis and DNA damage [43].

LYN, as a member of the Src family tyrosine kinases, regulates various cellular functions including proliferation, metabolism, differentiation, migration, and apoptosis [44]. Elevated LYN expression has been associated with enhanced tumor growth, metastasis, and poor prognosis in different cancer types [[44], [45], [46], [47]]. Several studies have reported that LYN is frequently overexpressed in BC cells, particularly in TNBC [48,49]. Pénzes K et al. (2014) demonstrated that knockdown of LYN significantly inhibited the migration of TNBC cells [50]. Tornillo G et al. (2018) introduced LYN as an oncogene driver that stimulates cell migration and invasion in aggressive TNBC cells [51]. Egeland EV et al. (2024) revealed that LYN is upregulated in paclitaxel-resistant TNBC patients, suggesting its potential as a therapeutic target in chemotherapy-resistant patients [52]. Chaudhary N et al. (2025) observed that LYN contributes to a hyperphosphorylation state of the EGFR-SRC axis in proliferating, drug-tolerant, persistent TNBC cells, leading to downstream overactivation of STAT3, AKT, and MAPK pathways associated with tumor cell growth, survival, and therapeutic resistance [53].

Human X-box binding protein-1 (XBP1) is an alternatively spliced transcription factor 1(XBP1s) involved in the unfolded protein response (UPR), a stress-signaling pathway that regulates cell survival and apoptosis [54]. Several studies demonstrated that overexpression of XBP1s is associated with reduced sensitivity to endocrine therapy, estrogen-independent growth, and poor outcomes in ER-positive BC tumors [55,56]. Ming J et al. (2015) showed that inhibition of XBP1expression using STF-083010 (an XBP1 splicing inhibitor) restored endocrine sensitivity in tamoxifen-resistant MCF7 tumors. Furthermore, they found that XBP1 expression is highly correlated with overall survival in the ER-positive BC tumors, but not in other ER-negative tumors [57]. In TNBC cell lines, a study from Chen X et al. (2014) reported that XBP1s promotes oncogenesis in TNBC through transcriptionally cooperating with the hypoxia-responsive transcription factor HIF-1α [58]. Yang S et al. (2022) reported that XBP1 exerts a dual and context-dependent effect in human TNBC cell lines and xenograft models. They observed that overexpression of XBP1 can inhibit tumor growth through interaction with the Hippo/YAP signaling, while promoting migration and cell invasion via the JNK/EMT pathway [59]. Our results revealed a significant downregulation of XBP1 in TNBC tissues compared to other BC subtypes and normal breast tissues. This downregulation may reflect loss of growth-regulatory functions in specific TNBC subtypes or an adaptive mechanism to evade UPR-induced apoptosis. These observations identify the importance of further studies into the context-dependent or isoform-specific roles of XBP1 in heterogeneity and therapeutic response of TNBC.

In terms of DEMs validation, among upregulated DEMs, the expression level of hsa-miR-16-2-3p was notably higher in TNBC samples, compared to non-TNBC subtypes and normal samples. Among downregulated DEMs, hsa-miR-5684 showed a significantly reduced expression in TNBC samples compared to other groups. The overexpression of circulating miR-16-2-3p has been proposed as a potential diagnostic biomarker in patients with hepatocellular carcinoma, non-syndromic cleft lip (NSCL), diabetes mellitus-associated coronary microvascular dysfunction, and multiple sclerosis [[60], [61], [62], [63]]. Zou J et al. (2016) reported that upregulation of miR-16-2-3p in NSCL patients compared to control samples might be involved in the progression of this disease [61]. Similarly, Han T et al. (2019) established that overexpression of miR-16-2-3p in cleft lip tissues and maxillary primordium mesenchymal cells (MPMCs) may promote apoptosis and suppress cell proliferation and migration through repression of the PDPK1/AKT signaling pathway. Collectively, their findings suggested miR-16-2-3p as a promising target for the treatment of NSCL [64]. Liu Y et al. (2024) showed that overexpression of cardiac miR-16-2-3p can alleviate coronary microvascular dysfunction in diabetes by modulating the fatty acid degradation pathway in endothelial cells. They proposed miR-16-2-3p as a potential therapeutic target in cardiovascular diabetology. [62]. However, downregulation of miR-16-2-3p has been found in mandibular prognathism and osteosarcoma patients. Maximov VV et al. (2019) demonstrated that reduced expression of miR-16-2-3p promoted tumor growth, metastasis, and chemoresistance in osteosarcoma cells by directly targeting the FGFR2 pathway [65]. Our results also indicated a significant upregulation of miR-16-2-3p in TNBC tissues compared to other BC subtypes and normal cells. These observations support a tissue-type and context-dependent role for miR-16-2-3p and its identifications in aggressive and drug-resistant highlights its potential as a novel biomarker.

hsa -miR-5684 was identified as a predictive biomarker for axitinib response in Asian patients with metastatic advanced hepatocellular carcinoma [66]. Zhang Z et al. (2020) reported that exosomal miR-5684 is significantly downregulated in non-small cell lung cancer and may serve as a diagnostic and prognostic biomarker [67].

In our study, miR-5684 was also significantly downregulated in TNBC tissues compared to other BC subtypes and normal samples. These findings suggest that hsa -miR-5684 may represent a potential biomarker for TNBC diagnosis and therapy, although further studies are needed to validate its clinical relevance.

All findings of this study are based on publicly available datasets and in silico analyses, without experimental validation. In addition, the included GEO datasets differ in sample characteristics, platforms, and experimental conditions, which may introduce variability across studies.

## 5 Conclusion

This study identified a novel mRNA-miRNA interaction network in aggressive and chemoresistant TNBC samples. Using bioinformatics analysis, we identified miR-4690-5p and miR-338-3p as key regulatory miRNAs. In silico validation using UALCAN further supported that the BRD4 and LYN genes, along with miR-16-2-3p, were significantly overexpressed, whereases XBP1 gene and miR-5684 were markedly downregulated in TNBC samples. These findings suggest that these molecules may represent tissue-specific and context-dependent biomarkers, although further experimental studies are needed to clarity their molecular roles and underlying mechanisms.

## Ethics approval and consent to participate

Not applicable.

## Author contributions

All authors made substantial and meaningful contributions to the execution of this study.

MFD: Conceptualized and designed the research framework, curated datasets, performed data analysis and interpretation, drafted the original manuscript, and developed visual representations. ERB: Curated data, conducted analytical procedures and interpretation, contributed to data visualization, and revised the manuscript.SD: Conducted research, curated data, and participated in the initial analysis. MJ: Conducted research and curated data.BR: Provided input on study design and conceptualization, critically reviewed and revised the manuscript, and oversaw the overall research process. All authors reviewed and approved the final version of the manuscript for submission.

## Funding

No funding or sponsorship was received for this study or the publication of this article.

## Declaration of competing interest

The authors report no conflict of interest.

## Data Availability

Data will be made available on request.
